# Simultaneous Harvesting of Bipolar Plasmonic Hot Carriers for Boosting Photoconductivity in Ag Nanoprism‐Coupled Lateral Si *p–n* Junction

**DOI:** 10.1002/advs.202414654

**Published:** 2025-02-28

**Authors:** Yujin Park, Jihyang Park, Yeonghoon Jin, Yujin Roh, Hyunhwa Lee, Kyoungsik Yu, Moonsang Lee, Jeong Young Park

**Affiliations:** ^1^ Department of Chemistry Korea Advanced Institute of Science and Technology (KAIST) Daejeon Yuseong‐Gu 34141 Republic of Korea; ^2^ Department of Materials Science and Engineering Inha University 100 Inha‐ro, Incheon Michuhol‐Gu 22212 Republic of Korea; ^3^ Program in Semiconductor Convergence Inha University 100 Inha‐ro, Incheon Michuhol‐Gu 22212 Republic of Korea; ^4^ School of Electrical Engineering Korea Advanced Institute of Science and Technology (KAIST) Daejeon Yuseong‐Gu 34141 Republic of Korea

**Keywords:** Ag nanoprism, hot electrons, hot holes, localized surface plasmon resonance, plasmonic photodetector

## Abstract

Plasmonic hot carriers have garnered considerable attention in photovoltaics and photocatalysis, yet their full potential is limited by the challenge of harvesting both positive and negative polarity hot carriers at the same time. Here, an unprecedented plasmonic hot carrier device capable of extracting both types of hot carriers simultaneously is demonstrated. This scheme involves generating and harnessing plasmonic hot electrons and holes concurrently using a lateral Si *p–n* junction diode coupled to Ag nanoprisms. The experimental and numerical results jointly reveal precise control of the generation and injection of plasmonic hot carriers, stemming from differing injection probabilities of each type of hot carrier into the substrates. It is shown that the bipolar plasmonic photodetector exhibits outstanding performance compared to plasmonic devices utilizing single‐polarity hot carriers, attributed to the simultaneous participation of plasmonic hot carriers in the photoconductivity nature of the diode. It is believed that this strategy of harnessing bipolar hot carriers will pave the way for the rational design of future plasmonic applications by providing significantly improved photoconductivity and flexible utilization of hot carriers.

## Graphical abstract

This study demonstrates a plasmonic hot carrier device capable of extracting both types of hot carriers simultaneously using a lateral Si *p–n* junction diode coupled to Ag nanoprisms. The experimental and numerical results jointly reveal precise control of the generation and injection of plasmonic hot carriers, stemming from differing injection probabilities of each type of hot carrier into the substrates.

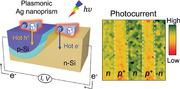

## 1 Introduction

Plasmonic hot carriers (HCs), consisting of highly energetic electrons and holes out of thermal equilibrium states in metallic nanostructures through Landau damping, can reshape the future of various technologies and engineering fields, including photochemistry, energy harvesting, and optoelectronics.^[^ ^1^, ^2^, ^3^, ^4^, ^5^, ^6^ ^]^ Despite the virtues of HCs with high kinetic energies of 1–3 eV, their fast‐disappearing nature with ultrashort lifetime and mean‐free path (I_mfp_) hinder efficient performance, constraining their widespread practical utilization.^[^ ^7^, ^8^, ^9^ ^]^ In this regard, it is essential to understand the interplay between environmental changes affecting plasmonic architecture and their impact on device performance to fully exploit the unique benefits provided by both hot electrons and hot holes. Recent investigations have underscored the potential of judicious selection of the plasmonic environments for reforming the HC population and transfer efficiency, thereby enabling improved HC harvesting.^[^ ^10^, ^11^, ^12^, ^13^ ^]^ For example, the asymmetries in the energy and density distribution of HCs result in distinct optimal operating spectral ranges for hot electrons and hot holes.^[^ ^14^, ^15^, ^16^ ^]^ Generally, hot electrons exhibit a longer I_mfp_, thereby a higher injection probability at high carrier energies (>1 eV) compared to hot holes. As a result, they have found optimized applications for harvesting visible light.^[^ ^17^, ^18^, ^19^, ^20^, ^21^ ^]^ Meanwhile, hot holes typically indicate a longer I_mfp_ at a lower energy regime (<1 eV), rendering them suitable for detecting or harnessing the infrared spectrum.^[^ ^22^, ^23^, ^24^, ^25^ ^]^ Notwithstanding the apparent and complementary advantages of individual HC polarities with differing dynamics and energetics across various wavelength regimes, most research has been devoted to extracting only either hot electrons or hot holes with a single polarity.^[^ ^26^, ^27^, ^28^, ^29^, ^30^, ^31^, ^32^ ^]^ This limitation primarily lies in the challenge of establishing energy‐selective contacts capable of collecting both HCs. Although a few studies have addressed simultaneous HC collection,^[^ ^33^, ^34^, ^35^, ^36^ ^]^ in situ observations of HC flow and their energy distribution remain elusive. Moreover, since HCs within a single pair affect each other's dynamics,^[^ ^8^, ^37^ ^]^ conventional HC‐device architectures where both hot‐electron‐ and hot‐hole injections occur at the same location are not suitable for independently assessing the distinct injection processes. This limited in‐depth exploration of the simultaneous harvesting of bipolar HCs has impeded a deeper understanding of the underlying physics governing the injection process of individual HC polarities within a single device. More specifically, the comparative generation and transport characteristics of HCs under identical conditions or external stimuli (e.g., light irradiation or applied bias) remain poorly understood, thereby limiting their potential for utilization in desirable applications. Therefore, direct experimental exploration of the concurrent generation and harvesting of plasmonic HCs is essential for gaining a deeper insight into their fundamental nature and for rendering highly efficient futuristic plasmonic device platforms.

Here, we introduce an innovative plasmonic platform that leverages HCs with two different polarities, designed to shed light on their underlying injection process through direct in situ visualization of HC flux. This platform integrates plasmonic Ag nanoprism arrays with a lateral Si *p–n* junction diode capable of creating energy‐selective contact interfaces. These interfaces enable the simultaneous collection of localized surface plasmon resonance (LSPR)‐driven HCs with opposing polarities by simply tuning the Fermi level of the Si substrate. To scrutinize the injection process of each HC polarity independently, we employ a lateral Si *p–n* junction diode specifically optimized to examine the injection process of each HC in distinct spatial regions. The injection behavior of each plasmonic HCs is explored using high spatial resolution photocurrent maps, which reveal asymmetrical HC injection into the Si platform. Moreover, we observe this asymmetry can be effectively regulated with external bias. These experimental findings are further corroborated by our theoretical calculation, which reveals the asymmetrical energy distribution of hot electrons and hot holes. Last, we show that this bipolar plasmonic architecture substantially enhances photodetection performance compared to plasmonic HC devices utilizing single‐polarity HCs, attributed to the combined effects of bipolar plasmonic HC injection and near‐field enhancement (NFE), thereby validating the efficacy and controllability of bipolar plasmonic HC flow. We believe that this study provides deeper insights into manipulating the nature of HCs with opposing polarities and offers a strategic framework for designing future LSPR‐based HC devices with the flexibility to utilize HCs of differing polarities.

## 2 Results and Discussion

### 2.1 Characterizing Ag Nanoprisms/Lateral Si *p–* *n* Junction Structure

**Figure 1:**
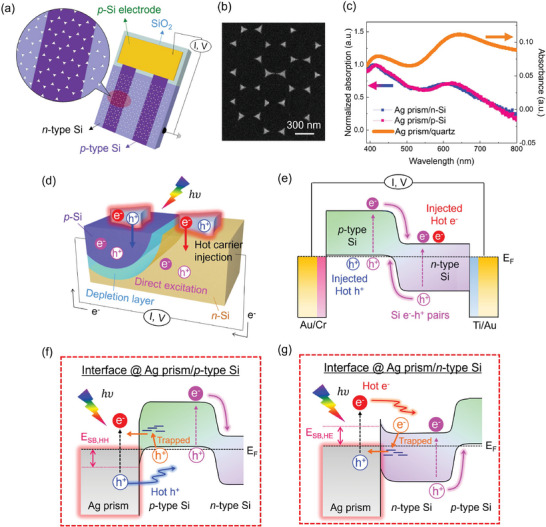
Structure of the Ag prism/lateral Si *p–n* junction. a) Scheme of a plasmonic architecture consisting of Ag nanoprisms deposited on a lateral Si *p–n* junction. b) SEM image showcasing the active area of an Ag prism/lateral Si *p–n* junction. c) Absorption spectra of Ag prism/*n*‐Si and Ag prism/*p*‐Si, overlaid with that of Ag prism/quartz. d) Schematic diagram and e) energy band diagram illustrating photocurrent generation mechanism involving HC injection and DE in the Ag prism/lateral Si *p–n* junction. Mechanism of HC injection and charge neutrality maintenance at the f) Ag prism/*p*‐Si interface and g) Ag prism/*n*‐Si interface.

To establish the platform to extract hot electrons and hot holes simultaneously, we constructed a bipolar plasmonic architecture consisting of Ag nanoprisms/lateral Si *p–n* junction (**Figure** **1**a). Detailed fabrication procedure is provided in the Methods section. The diode comprises *p*‐type Si (*p*‐Si) stripes patterned on an *n*‐type Si (*n*‐Si) substrate, thus resulting in alternating *p*‐Si and *n*‐Si districts. The *p*‐Si and *n*‐Si regions in a Si substrate function as supporting materials below the plasmonic Ag nanoantenna to collect plasmonic hot holes and hot electrons, respectively. By introducing dopants into selective areas of the *n‐*Si substrate, we were able to locally modulate its electrical conductivity polarity and work function. This made the Si *p‐n* junction template suitable for the simultaneous extraction of hot electrons and holes under light irradiation. The average doping concentrations of *p*‐Si and *n*‐Si were estimated to be 1.3 × 10^19^ and 1.7 × 10^14^ cm^−3^, confirming the work functions of *p‐*Si and *n‐*Si to be 5.3 and 4.4 eV, respectively (Figure S1 and See Note S1, Supporting Information). Additionally, we implemented Kelvin probe force microscopy (KPFM) to clarify the successful operation of the bare Si *p–n* junction diode. It is apparent that the depletion width of the diode widened toward the *n‐*Si region from 2 to 8 µm with increasing the reverse bias (Figure S2, Supporting Information). We also calculated the depletion width based on depletion approximation and Poisson's equation, which was consistent with the experimental results (See Note S2, Supporting Information). These results reflect that the bare lateral Si *p–n* junction was successfully established. Furthermore, both *n*‐Si and *p*‐Si territories exhibited smooth surfaces with a height variation of less than 1 nm, demonstrating that the supporting Si *p–n* junction template is suitable for constructing the plasmonic architecture within identical geometric environments (Figure S3, Supporting Information). Subsequently, the hexagonal arrays of triangular Ag nanoprisms, each with a height of 25 nm and a width of 170 nm, were patterned onto the Si *p–n* junction using nanosphere lithography method (Figure 1b; Figure **S4**, Supporting Information).^[^ ^26^, ^27^, ^28^ ^]^ We selected triangular Ag nanoprisms to maximize hot spots at their corners and edges, enhancing the LSPR effects. To ensure an unimpeded HC injection process, we employed nanosphere lithography for fabricating plasmonic nanoantenna instead of using bottom‐up synthesized nanoparticles, avoiding any interference from insulating capping ligands. To elucidate the LSPR wavelength of the device, the absorption spectra of the platform were examined (Figure 1c). Bare Si substrates have a monotonous change with few visible peaks, (Figure S5, Supporting Information) while Ag prism/*n*‐Si and Ag prism/*p*‐Si show two distinct LSPR absorption plateaus, centered at ≈420 and 640 nm, aligning with the absorbance spectrum of Ag prism/quartz. These observations indicate that the Ag nanoprism/Si *p–n* junction can serve as an effective solar energy converter, leveraging strong absorption at the LSPR wavelength peaks. We further examined the creation of Schottky contact between individual Ag nanoprisms and Si substrates by measuring the current–voltage (*I–V*) curves for Ag prism/*p*‐Si and Ag prism/*n*‐Si using a conductive atomic force microscope (c‐AFM) (Figure S6, Supporting Information). The results clearly exhibit rectifying behavior, indicating well‐established Schottky junctions between Ag nanoaprisms and the supporting *p*‐Si or *n*‐Si materials. By fitting the *I–V* curves to the thermionic emission equation,^[^ ^38^ ^]^ the Schottky barrier heights (E_SB_) between Ag nanoprisms and *p*‐Si (E_SB,HH_) and *n*‐Si (E_SB,HE_) were estimated to be −0.60 and +0.53 eV with respect to the Ag Fermi level, respectively. This suggests that only hot carriers exceeding the E_SB_ can flow into the supporting materials to generate plasmonic photocurrent. To elucidate the nature of photocurrent generated by HCs with opposite charges in the Ag nanoprisms/Si *p–n* junction, we illustrate the operational principle and energy band diagram, as shown in Figure 1d,e. In the bare Si *p–n* junction, illumination with sufficiently energetic light generates photocurrent by creating electron‐hole (e^−^–h^+^) pairs through direct excitation (DE) within the Si. After loading Ag nanoprisms onto the Si *p–n* junction diode, the photocurrent is influenced not only by DE but also by the combined effects of NFE and plasmonic HCs, derived from LSPR. Notably, the NFE contributes to further enhancing DE within the Si *p–n* junction. In parallel, since the Ag prism/*n*‐Si (Ag prism/*p*‐Si) interface produces upward (downward) band bending, hot electrons (hot holes) are collected in the *n*‐Si (*p*‐Si) region, followed by charge drift in the depletion region, as shown in Figure 1e. Incidentally, the hot electrons (hot holes) may remain in the Ag prism after hot holes (hot electrons) are injected into the *p*‐Si (*n*‐Si) substrate. While depletion of the Ag nanoprism after HC injection and its impact on the Schottky interface may occur, the Ag/Si junction continued to display stable Schottky behavior and current flow even after several minutes of scanning. This indicates that the charge loss was effectively compensated by the circuit. We propose that this charge imbalance is compensated by the low‐energy charges,^[^ ^39^, ^40^ ^]^ with the extrinsic carriers at the valence (conduction) band edge in *p*‐Si (*n*‐Si) substrate transferring into Ag prism through intra‐bandgap states in Si substrate (Figure 1f,g). Indeed, we observed the fingerprints of intra‐bandgap states in the Si substrates and charge‐trapping‐assisted photoconductivity enhancement in Ag prism/Si *p–n*, as discussed further down. Therefore, the overall photocurrent in the Ag prism/Si *p–n* junctions can be attributed to the combined effects of DE within the Si *p–n* junction, as well as the NFE and plasmonic HC injection from the LSPR nature.

### 2.2 Enhanced Photoconductivity on Plasmonic Ag Nanoprisms/Si *p–n* Junction

**Figure 2:**
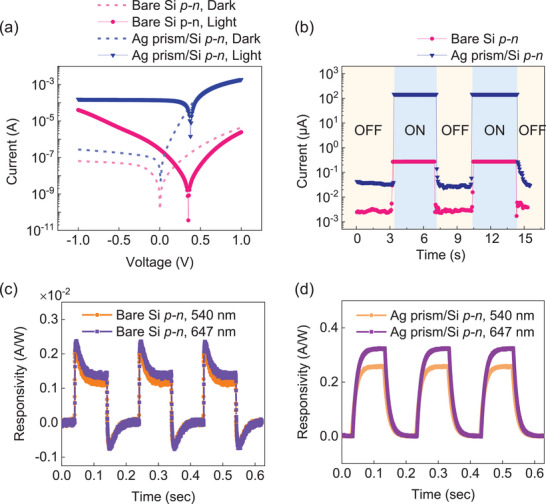
Characterization of photoconductivity enhancement in the Ag prism/lateral Si *p–n* junction. a) *I–V* curves and b) short‐circuit currents measured for both the bare Si *p–* *n* and the Ag prism/Si *p–* *n* photodiodes. A tungsten‐halogen lamp with an intensity of 9 mW cm^−2^ was used as the light source. Transient responsivity was measured on c) the bare Si *p–n* and d) the Ag prism/Si *p–n* junction under the wavelengths of 540 and 647 nm without external bias.

**Figure** **2** presents the opto‐electrical characteristics of the bare and the plasmonic Si *p–n* junction diodes. Both diodes exhibited clear rectifying behaviors without light illumination (Figure 2a). The stability of the Ag prism/Si *p–n* device was confirmed by comparing *I–V* curves measured immediately after fabrication and after 24 h, which demonstrated negligible changes under both dark and light conditions, indicating insignificant degradation of the Ag prisms (Figure S7, Supporting Information). We observed a higher dark current level in the plasmonic Si *p–n* junction compared to the bare device across the entire bias range. Furthermore, reducing the spatial ratio of Ag prisms by 50% led to a 90% decrease in the dark current (Figure S8, Supporting Information), suggesting the internal electric field (*E*‐field) resulting from the Schottky interface at Ag prism/Si may be the cause.^[^ ^41^, ^42^ ^]^ Upon light illumination, both devices showed enhanced current values, compared to those under dark conditions. We observe a logarithmic increase in current in the reverse bias region on the bare Si *p–n* junction, attributed to carrier generation at the recombination‐generation center arising from intra‐bandgap states within the depletion layer, known as Shockley–Read–Hall generation.^[^ ^43^, ^44^ ^]^ It is crucial to highlight that the plasmonic platform yielded ≈543 times higher photocurrent than that of the bare *p–n* diodes. This notable increase can be readily attributed to the NFE and plasmonic HC injection from Ag nanoprisms. Furthermore, the open‐circuit voltages of the bare and the plasmonic diode shifted to ≈+ 0.35 and + 0.38 V, respectively, indicating that the introduction of plasmonic environments enhanced the photovoltaic effect. Figure 2b displays the dynamic short‐circuit current of the bare and the plasmonic *p–n* junction diode, both of which exhibited immediate and sharp response under illumination. To eliminate the contribution of the observed dark current enhancement after loading the Ag nanoprisms, we normalized it by comparing the light on/off current ratio. Notably, the on/off ratios for the bare and plasmonic Si *p–n* junctions are 99 and 4,455, respectively, highlighting the outstanding solar antenna capability of the plasmonic Si *p–n*. To further elucidate the plasmonic photoresponse characteristics, we investigated the transient photoresponsivity for the bare and the plasmonic Si *p–n* junction at different wavelengths. The bare Si *p–n* junction had a photoresponsivity of 0.0025 A/W, showing minimal variation across wavelengths (Figure 2c). In contrast, the plasmonic Ag nanoprisms/ Si *p–n* junction displayed a dramatic increase in photoresponsivity of 0.25 and 0.33 A/W at 532 and 647 nm, respectively, indicating the plasmonic nature significantly promoted photoconductivity (Figure 2d). The rise and fall times of the diodes, defined as the time interval for the current rise from 10% to 90% of the peak value and vice versa, are summarized in Figure S9 and Table S1 (Supporting Information). The bare Si *p–n* junction had a rise/fall time of 0.4/0.8 ms regardless of the wavelength. In contrast, the Ag prism/Si *p–n* junction displayed slower rise/fall times of 28.6/28.8 ms at 532 nm, with even slower times of 29/28.9 ms at the LSPR wavelength of 647 nm. We submit that the slower photocurrent response in the plasmonic Si *p–n* junction indicates a photoconductive gating effect, where extrinsic carriers within the Si substrate are trapped in the intra‐bandgap states at Ag prisms/Si interface and migrate to the depleted Ag prisms to restore them. In the same context, the slower rise/fall times under LSPR wavelengths suggest that the facilitated HC injection takes more time to restore the depleted Ag prisms. Incidentally, since HC injection typically occurs within 200 fs,^[^ ^8^, ^45^ ^]^ we infer HC injection occurs first, followed by the slower transient response (≈29 ms), which is associated with the trapping of extrinsic carriers in the Si substrates’ intragap states.^[^ ^46^ ^]^

### 2.3 Nanoscopic Investigation of Hot‐Carrier Flow at Hot Spots

**Figure 3:**
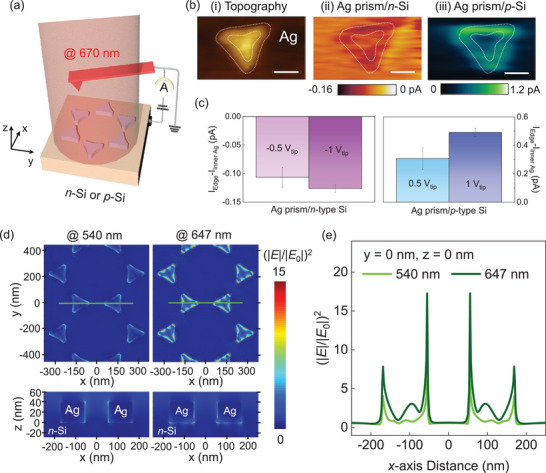
Nanometer‐scale current mapping images of the Ag nanoprism/lateral Si *p–n* junction. a) Schematic showing photocurrent mapping measurement using pc‐AFM on the Ag prism/Si *p–n* junction. b) i) Topography and a representative photocurrent mapping image of ii) Ag prism/n‐Si and iii) Ag prism/p‐Si. The scale bars are 80 nm. The white dashed lines indicate the boundaries of the edges and inner side of Ag prism. c) Photocurrent difference between the edges and inner side of the Ag prism under reverse bias for Ag prism/n‐Si (left) and Ag prism/p‐Si structure (right). d) FDTD simulations of E‐field intensity (|E|/|E0|)^2^ on Ag prism/n‐Si under 540 and 647 nm. Simulations are shown in a top‐view (upper panel) and a cross‐sectional view along the green solid line in the top‐view image (lower panel). e) E‐field enhancement intensity profile along the *x*‐axis at y = 0 nm and z = 0 nm of the Ag prism/n‐Si structure.

To further investigate the plasmonic bipolar‐HC behaviors at the hot spots, we mapped the photocurrent distribution using photoconductive atomic force microscopy (pc‐AFM) under incident laser wavelengths of 670 nm with a power intensity of 0.4 W cm^−2^ (**Figure** **3**a). The structure of the Ag nanoprism/Si supports constructed a complete plasmonic Schottky nanodiode, providing a framework to achieve conclusive evidence of hot electrons and holes generated through LSPR excitation on a single Ag prism. Upon HC generation on a single Ag nanoprism, HCs with sufficient kinetic energy will be transferred across the Schottky barrier, whereas those with insufficient energy to overcome the barrier will be redirected back to the AFM tip, completing the global circuit. The plasmonic charge transfer near the tip enables the mapping of photocurrents at the hot spots with the nanoscale spatial resolution. As shown in Figure 3b, the photocurrent maps clearly demonstrate the pronounced photocurrents at the edges of the Ag prism compared to those at the interior. This is because, with the occurrence of the LSPR effect, oscillating free electrons tend to accumulate at the edges of the Ag prism. Consequently, this leads to an intensified localized *E‐*field at the edges of the nanoprism and an amplified HC‐driven photocurrent. Notably, the direction of the photocurrent differs between the two structures, where the Ag prism/*n*‐Si indicates a negative photocurrent, while the Ag prism/*p*‐Si structure shows a positive photocurrent (Figure S10, Supporting Information). These opposite charge flows explain the collection of different types of hot carriers in *n*‐Si and *p*‐Si regions, supporting the notion that hot holes are the primary contributor in the *p*‐Si region, while hot electrons are more significant in the *n*‐Si region. To assess the viability of effective and customizable extraction of the plasmonic HCs, we applied the reverse bias between the Ag nanoprism and the *n*‐Si (or *p*‐Si) to reduce the E_SB,HE_ (or E_SB,HH_) (Figure 3c). It demonstrates that the photocurrent difference between the edges and the interior of the Ag prism became more distinctive as the reverse bias increased, indicative of improved HC harvesting through the external bias. To explicitly interrogate the nature of the plasmonic HC flux amplification, the *E‐*field enhancement distribution of the Ag prism/*n*‐Si (Figure 3d) and the Ag prism/*p*‐Si (Figure S11, Supporting Information) was estimated using the finite‐difference time‐domain (FDTD) method. The confined *E*‐field intensity at 647 nm wavelength surpasses that at 540 nm, reflecting that the strong plasmonic resonant coupling was generated under an LSPR wavelength of 647 nm. Moreover, significant field confinement was observed at the edge site of the Ag nanoprisms under 647 nm wavelength illumination. This is more clearly shown in the *E‐*field profiles acquired at both Ag prism/*n*‐Si (Figure 3e) and Ag prism/*p*‐Si (Figure S11b, Supporting Information) interface along the *x*‐axis distance, with y = 0 and z = 0 nm. It is apparent that *E‐*field enhancements at the Ag prism edges increased by 1.9 under 647 nm incident light, compared to 540 nm for both Ag prism/*n*‐Si and Ag prism/*p*‐Si structures. These findings indicate that the formation of plasmonic hot spots at the edges leads to enhanced HC flux into the plasmonic platforms.

### 2.4 Quantitative Analysis of Bipolar Hot‐Carrier Flow in Photoconductivity Enhancement

**Figure 4:**
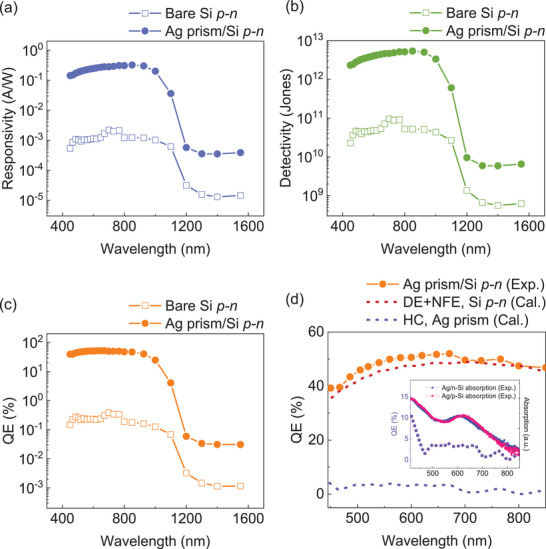
Characterization of photodetection performance of the Ag prism/lateral Si *p–n* junction. a) Responsivity, b) detectivity, and c) QE as a function of wavelength measured on the bare Si *p–n* and the Ag prism/Si *p–n* junction. d) Comparison of measured QE (orange circle symbol) and calculated QE. The red and purple dashed curves represent the theoretically estimated QE due to DE in Si *p–n* junction and HC injection from Ag nanoprims, respectively. The inset shows the comparison of calculated QE of HCs from Ag prisms, overlaid with the normalized absorption spectra of Ag prims on either *n*‐Si or *p*‐Si .

To validate the contributions of plasmonic bipolar HC nature in enhancing photoconductivity within the plasmonic Ag prism/Si *p–n* junction architecture, we inspected the photoresponse characteristics of the bare and plasmonic Si *p–n* junction diode from visible to near‐infrared spectrum (**Figure** **4**). The photoresponse characteristics, including responsivity, detectivity and quantum efficiency (QE), were calculated using the equations in Note S3 (Supporting Information). The photoresponse characteristics across the entire wavelength range showed a significant enhancement at <1100 nm, correlating to the Si bandgap of ≈1.1 eV. The photoresponse of the plasmonic Ag nanoprism/Si *p–n* junction platform was more than 100 times higher than that of the bare Si *p–n* junction in the wavelength range from 400 to 1100 nm, indicating that the NFE and plasmonic HC injection jointly govern photoconductivity. In contrast, the photodetection characteristics for wavelengths longer than 1100 nm in the plasmonic diode increased by only a factor of 10, compared to the bare Si *p–n* diode, indicating that its photoconductivity is dominated by the NFE, with negligible contributions from the DE or HC injection. Furthermore, we compared the photodetection performance of our plasmonic architecture with other plasmonic HC devices utilizing single‐polarity HCs (See **Table** **1**). Remarkably, our proposed plasmonic bipolar‐HC platform exhibited outstanding photodetection performance across all metrics without external bias, surpassing single‐polarity plasmonic HC photodetectors reported in the earlier literature. Particularly, our plasmonic platform showcases substantial responsivity enhancement, even exceeding the values achieved with external bias, highlighting the efficacy of bipolar HC harvesting in boosting photoconductivity. To gain deeper insight into the contribution of bipolar HC injection in improving photoconductive nature, we estimate the theoretical contribution of HCs from the total QE (Figure 4d). We calculated the current flow within the Si *p–n* substrate of the Ag prism/Si *p–n* device, considering it as the combination of DE and NFE, and then subtracted this value from the total photocurrent to assess the contribution of HCs (See Note S4, Supporting Information). Consequently, we determined that the QE for the combined contributions of DE and NFE in the plasmonic diode is ≈47.9%, while the QE of HC injection is ≈3.4% at the LSPR absorption band, corresponding to 93.4% and 6.6% of the total QE, respectively. Since the plasmonic absorption band spectrally overlaps with the Si substrate's absorption range, the QE enhancement is predominantly driven by NFE, with a smaller contribution from HC injection.^[^ ^47^, ^48^ ^]^ Notably, the contour of the QE dominated by bipolar HC injection closely mirrors the trajectory of the absorption spectra of the Ag/Si structure, as represented in the inset of Figure 4d. This close correlation between the plasmonic absorption peak and QE peak verifies that the nature of the plasmonic bipolar HCs dictates the light absorption properties of the diodes. Furthermore, considering the typical QEs of plasmonic Schottky devices read below 1% for hot‐electron collection ^[^ ^49^, ^50^, ^51^ ^]^ and below 3% for hot‐hole collection,^[^ ^9^, ^15^, ^52^ ^]^ the observed 3.4% QE for bipolar HC extraction in the Ag prism/Si *p–n* junction, surpassing the QEs of other single‐polarity HC applications, underscores the effectiveness of bipolar HC harvesting in improving QEs.

**Table 1:** Comparison of the figure‐of‐merits of the current Ag prism/Si *p–n* photodetector with those of other plasmonic photodetectors.

Device	Measurement conditions		Figure‐of‐merits				Ref
	Bias [V]	Wavelength [nm]	Responsivity [A/W]	Responsivity enhancement^a)^	Detectivity [Jones]	QE [%]	
Ag prism/Si *p–n*	0	647	269 × 10^−3^	217.4	4.41 × 10^−12^	52	This work
Au prism/*n*‐InSe	0	685	244 × 10^−3^	12	3.35 × 10^−12^	<10	[29]
Porous Ag/TiO_2_	0	450	7.4 × 10^−3^	NA	5.32 × 10^−10^	0.91	[21]
Au grating/*p*‐Si	0	1548	13 × 10^−3^	NA	NA	6	[25]
Au/*n*‐Si NW	1.5	1150	94.5 × 10^−3^	< 9.5	4.38 × 10^−11^	11.7	[30]
Au@MoS_2_/*p*‐Si	4	300–800	10‐30	27.3	NA	NA	[31]
Plasmonic Al/*n*‐GaN	−5	355	670	10	1.48 × 10^−15^	NA	[32]

^a)^ Responsivity Enhancement = (Responsivity with plasmonic antenna) / (Responsivity without plasmonic antenna).

### 2.5 Investigation of Asymmetric Injection Probabilities of Hot Electrons and Hot Holes

**Figure 5:**
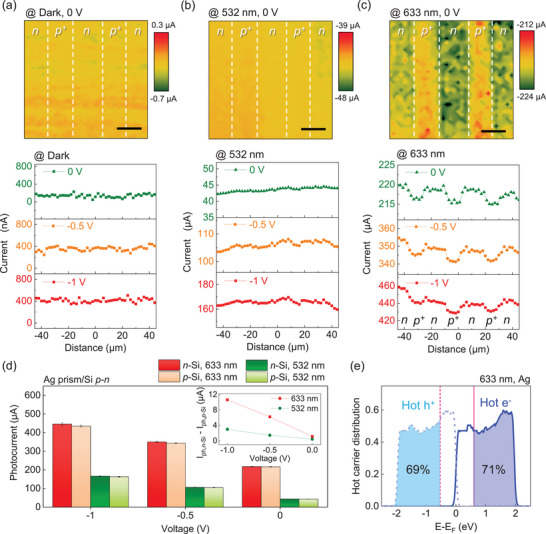
Micrometer‐scale current mapping images of the Ag prism/lateral Si *p–n* junction. Scanning current mapping images of the Ag prism/Si *p‐n* junction without any external bias (upper panel) and average current profiles acquired from scanning current maps measured on the identical device with an external bias varying from −1 to 0 V (lower panel) taken under laser wavelengths of a) dark conditions, b) 532 nm, and c) 633 nm. The scale bars in the images are 13 µm. The power density of the lasers was 76 mW cm^−2^. d) Comparison of photocurrents measured on the *n*‐Si and the *p*‐Si regions as a function of applied voltage under irradiation of 633 and 532 nm on the Ag prism/Si *p–n* junction. The inset shows the photocurrent difference between the *n*‐Si and the *p*‐Si regions as a function of applied voltage. e) Initial energy distribution of hot holes (sky blue dashed line) and hot electrons (blue solid line) upon excitation with 633 nm in Ag prisms. The E_SB_ for Ag/*p‐*Si (0.53 eV) and Ag/*n‐*Si (0.60 eV) are denoted by the dashed pink line and solid pink line, respectively. The shaded areas indicate the portion of HCs with energy beyond the Schottky barrier heights.

To visualize the bipolar HC‐injection‐driven photoconductivity enhancement, we obtained the macroscopic photocurrent distribution for both the bare and plasmonic Ag prism/Si *p–n* junctions. The photocurrent maps were obtained by measuring the current while scanning a continuous laser source with a 1 µm diameter across the surface of the diodes. The upper panels of **Figure** **5**a–c show the photocurrent maps under illumination at various wavelengths without external bias. The lower panels display the averaged absolute photocurrent profiles obtained from the current maps under applying a reverse bias between *n*‐Si and *p*‐Si range from −1 to 0 V (Figure S12, Supporting Information). We observed nearly constant current values across the entire device regions for the bare Si *p–n* diode, regardless of the light wavelength (Figure S13, Supporting Information). Additionally, there is insignificant photocurrent contrast in the Ag prism/Si *p–n* diode under dark and 532 nm, as presented in the upper panels of Figure 5a,b, which reflects that the DE is equivalent in both *n*‐Si and *p*‐Si territories within the Ag prism/Si *p–n* structure. In sharp contrast, the plasmonic diode showed a significant current contrast between *p*‐Si and *n*‐Si districts at 633 nm, indicating that the LSPR phenomenon governs the photoconductivity in the device. Figure 5d shows photocurrents obtained from *p*‐Si (I_ph,_ *_n_*_‐Si_) and *n*‐Si (I_ph,_ *_n_*_‐Si_) regions, with the photocurrent difference between *p*‐Si and *n‐*Si (I_ph,_ *_n_*_‐Si_–I_ph,_ *_p_*_‐Si_) summarized in the inset. Interestingly, a more substantial photocurrent contrast between *p*‐Si and *n*‐Si was monitored with increasing reverse bias, whereas the contrast was still negligible under dark and 532 nm conditions. To provide a better understanding of the observed photocurrent contrast, the injection process of hot electrons and hot holes in the Ag prism/Si *p–n* junction under external bias is explained in Figure S14 (Supporting Information). As the reverse bias increases, the energy levels of *n‐*Si are shifted to the lower levels, leading to the reducing the E_SB,HE_ between the Ag prism and *n‐*Si. This facilitates the hot‐electron injection across the Ag nanoprisms/*n‐*Si region, while the injection of hot holes at Ag prism/*p‐*Si remains constant. Conversely, the photocurrent difference disappeared with the application of forward bias on the plasmonic diode. We consider that the diffusion current of majority carriers in Si *p–n* junctions conceals the contribution of HC injection under forward bias.

Incidentally, the higher photocurrent in *n*‐Si compared to *p*‐Si may be due to differences in the DE within the Si substrate. However, we experimentally confirmed identical DE probabilities in both regions, as evidenced by negligible current contrast at 532 nm. We speculate that the DE remains identical across the *n*‐Si and *p*‐Si regions even under LSPR excitation, due to similar *E*‐field intensities at the hot spots, which result from the equivalent refractive indices of two Si substrates in the visible spectrum (Figure S15, Supporting Information). Indeed, our FDTD simulations demonstrated a 1.9‐fold increase in the averaged *E*‐field intensity at the hot spots for both Ag prism/*n*‐Si and Ag prism/*p*‐Si at 647 nm compared to 540 nm. In this regard, we attribute the observed photocurrent contrast to the different injection probability of plasmonic hot electrons and holes into the Si supports, with hot‐electron injection into *n*‐Si likely exceeding hot‐hole injection into *p*‐Si. To validate this hypothesis, we theoretically estimated HC injection probability into both *n*‐Si and *p*‐Si supports using the energy distribution of the join density of states (EDJDOS).^[^ ^11^ ^]^ Figure 5e illustrates the calculated initial energy distribution of HCs in Ag nanoprisms at 633 nm. It is well established that the probability of HC injection is proportional to the energy distribution of HCs exceeding the E_SB_ in metal–semiconductor structure. Based on the results, the injection probabilities of hot electrons and holes into the *n*‐Si and the *p*‐Si supports are 70.98% and 69.03%, respectively, supporting the higher photocurrent in the *n*‐Si region compared to the *p‐*Si region of the plasmonic Si *p–n* junction. Consequently, these fingerprints highlight that plasmonic hot electrons and hot holes can be generated simultaneously with an asymmetric energy distribution, and the injection probability of HCs with a specific polarity can be modulated by applying external bias.

## 3 Conclusion

In summary, we successfully demonstrated highly enhanced photoconductivity on the Ag prism/lateral Si *p–n* junction by manipulating the nature of HCs with opposing polarities. The established Schottky junction between the Ag prisms and the lateral Si *p–n* junction effectively enables the spatial separation of hot electrons in the *n‐*Si region and hot holes in the *p‐*Si region, allowing independent examination of each HC injection process. This bipolar HC injection was confirmed through nanometer‐scale photocurrent maps measured using pc‐AFM, revealing active HC injection at the hot spots located at the edges of the Ag prism. The macroscopic photocurrent maps further visualized asymmetric probabilities of HC injection, as evidenced by the photocurrent contrast between *n‐*Si and *p‐*Si regions, supported by theoretically calculated HC energy distribution. Additionally, we demonstrated that the distinct bipolar HC injection can be reversibly modulated with external bias, highlighting the effective customization of extracting bipolar HCs. Remarkably, the proposed plasmonic architecture achieved exceptional photodetection performance, surpassing values reported for single‐polarity HC devices, owing to the participation of bipolar HCs in the photoconductivity. We believe this plasmonic architecture has the potential to pave the way for the development of plasmonic photovoltaics with high photoconductivity, offering flexibility in the use of HCs with two contrasting charges. While our device is primarily characterized as a photodetector, it also holds significant potential for advancing sophisticated plasmonic photocatalysts capable of simultaneously facilitating oxidation and reduction reactions. Moreover, we anticipate it will serve as a valuable playground for conducting comparative studies on the behaviors of hot electrons and hot holes.

## 4 Experimental Section

### Fabrication of Lateral Ag Prism/Si *p–n* Junction

To create a lateral Si *p–n* junction, an array of *p‐*Si stripes was fabricated on an *n‐*Si (100) substrate doped with phosphorus at a low concentration of 1.7 × 10^14^ cm^−3^. The *p‐*Si stripes were formed by implanting boron at 50 keV and annealed at 1050 °C for 30 min, resulting in a boron concentration of 3 × 10^19^ cm^−3^. The *p‐*Si stripe is 12 µm wide and 3000 µm long with a nominal depth of 0.52 µm, spaced at 30 µm intervals. The surface of the lateral Si *p–n* junction substrate was cleaned using the RCA method to remove organic residues and rinsed with buffered oxide etchant (NH_4_F:HF = 6:1, SAMCHUN) to remove native oxide. Ohmic electrodes were created for the *p*‐Si and *n‐*Si by depositing Cr(10 nm)/Au(80 nm) and Ti(50 nm)/Au(50 nm), respectively. Ag nanoprisms were fabricated on the lateral Si *p–n* junction substrate by nanosphere lithography technique using polystyrene (PS) latex beads. To prepare a PS self‐assembled monolayer, a PS precursor solution was prepared by mixing 10 wt.% PS in water (Sigma–Aldrich Inc.) and ethanol in a 1:1 volume ratio. After that, a slide glass was tilted and immersed in a vessel filled with water, and 200 µL of sodium dodecyl sulfate (Sigma–Aldrich Inc.) mixed with water at 2 wt.% was dropped on the surface of the slide glass and the water. Subsequently, 10 µL of PS precursor solution slowly flowed onto the slide glass to create a PS self‐assembled monolayer floating on the water surface. The formed PS monolayer was lifted off from the water and deposited on the lateral Si *p–n* junction substrate surface. After sufficiently drying in air, a 25 nm Ag film was deposited using an e‐beam evaporator. Finally, the PS monolayer was removed by ultrasonic treatment, and Ag nanoprisms arranged in a hexagonal shape were formed on the lateral *p–n* junction.

### Characterization

The morphology of the Ag prism/Si *p–n* junction device was analyzed using a scanning electron microscope (SEM, SU5000, Hitachi), while the absorption spectrum of the Ag prisms on the Si substrate and absorbance spectrum of Ag prisms on quartz were measured using a UV–vis spectrometer (UV 3600, Hitachi). For the opaque Si substrates, transmittance was assumed to be zero, with absorption defined as 1–reflectance. Meanwhile, the absorbance was calculated as –log(transmittance). The *n‐*Si and *p*‐Si doping concentrations were determined using time‐of‐flight secondary ion mass spectrometry (TOF‐SIMS, M6, IONTOF GmbH), and the built‐in voltage of the Si *p–n* junction was estimated using the Mott–Schottky plot, which was measured using a multichannel potentiostat equipped with electrochemical impedance spectroscopy (AMETEK Princeton Applied Research, VersaSTAT3) at a frequency of 1 kHz and an amplitude of 10 mV. To examine the spectral responsivity, detectivity, and QE at various wavelengths, a wide‐band photoresponsivity analyzer (TG‐01, Taegang Tech. Inc.) was used with light‐emitting diodes (450–671 nm) and halogen lamps (671–1550 nm) as light sources. Specific light wavelengths were collected using bandpass filters, and light intensities were calibrated using conventional Si photodetectors. Photocurrent mapping images were obtained using a photocurrent scanning system in photoluminescence spectroscopy (XperRam, Nanobase) combined with a source meter unit (Keithley 2612A). The *I–V* curves and photocurrent of the fabricated device were characterized using a source meter (Keithley 2400), with a tungsten‐halogen lamp providing an intensity of 9 mW cm^−2^ as the light source. Local information about the device with nanometer‐scale spatial resolution, including morphology, *I–V* curve, surface potential, and photocurrent mapping images, was acquired using pc‐AFM, which involves modified sample stage of a commercial AFM (Agilent 5500 and Park NX10) with a laser system (OBIS series, Coherent).

### Theoretical Calculation of Hot‐Carrier Energy Distribution

To calculate the energy distribution of HCs (*P_dis_*), the energy distribution of joint density of states (EDJDOS) was utillized. Considering the low incident photon energy in the study (less than 3 eV), EDJDOS was simplified as D(E−hυ)×D(E), where D(E−hυ) and *D*(*E*) represent electronic DOS before and after photon absorption. Thus, *P_dis_* is approximated by the following equation

(1) $$\begin{array}{c} P_{dis}\;\left(E_{m}\right)=\frac{D\left(E_{m}-h\upsilon \right)f\left(E_{m}-h\upsilon \right)D\left(E_{m}\right)\left\{1-f\left(E_{m}\right)\right\}}{\int_{-\infty }^{\infty }D\left(E_{m}-h\upsilon \right)f\left(E_{m}-h\upsilon \right)D\left(E_{m}\right)\left\{1-f\left(E_{m}\right)\right\}dE_{m}} \end{array}$$

where *E_m_* is the electron energy relative to Fermi level (*E_m_* =  *E* − *E_F_*) and *f*(*E*) is the Fermi–Dirac distribution.

### FDTD Simulations

The finite‐difference time‐domain (FDTD) method (Lumerical) was utilized to estimate the *E‐*field distribution. A model system was created, consisting of Ag nanoprisms arranged infinitely in a hexagonal pattern on either *n‐*Si or *p‐*Si, with a side length of 170 nm and a height of 25 nm. The unit cell of the hexagonal Ag prism array was designated as the simulation area. A plane wave source of 400–900 nm was utilized as the light source, and it was positioned to irradiate in the backward direction along the *z*‐axis so that that light could be directed toward the centerline of the unit cell of Ag nanoprism arrays. To represent the average near‐field enhancement for different incident light polarizations, the light was polarized both parallel and perpendicular relative to the edge of Ag prisms. The side planes of the simulation area were set to periodic, and the other planes were set to perfectly matched layers. A mesh with 2 nm spacing in the *x‐* and *y*‐axes and a 0.5 nm spacing in the *z*‐axis was utilized for the Ag nanoprism. The refractive index and extinction coefficient of Ag, *n‐*Si, and *p‐*Si used in the simulation were experimentally measured using a spectroscopic ellipsometer (M2000D, Woollam).

## Conflict of Interest

The authors declare no conflict of interest.

## Author Contributions

Y.P. designed the plasmonic Ag nanoprism/Si *p–n* junction diode and performed the photoelectrical measurements. J.P. contributed to photoelectrical measurements. Y.J. contributed to the EDJDOS calculation. Y.R. and H.L. contributed to the pc‐AFM experiment. K.Y., M.L., and J.Y.P. analyzed the experimental data. All the authors contributed to the preparation and revision of the manuscript.

## Supporting information



Supporting Information

## Data Availability

The data that support the findings of this study are available from the corresponding author upon reasonable request.
