# Supplementary material for: Simultaneous Harvesting of Bipolar Plasmonic Hot Carriers for Boosting Photoconductivity in Ag Nanoprism‐Coupled Lateral Si p–n Junction
Source: Adv Sci (Weinh). 2025 Feb 28;12(23):2414654. doi: 10.1002/advs.202414654 (PMC12199441; doi:10.1002/advs.202414654)
Supplement: Supplementary file 1 — Supporting Information [file ADVS-12-2414654-s001.pdf]

ADVANCED SCIENCE
Open Access

Supporting Information

for *Adv. Sci.*, DOI 10.1002/advs.202414654

Simultaneous Harvesting of Bipolar Plasmonic Hot Carriers for Boosting Photoconductivity in Ag Nanoprism-Coupled Lateral Si *p–n* Junction

Yujin Park, Jihyang Park, Yeonghoon Jin, Yujin Roh, Hyunhwa Lee, Kyoungsik Yu, Moonsang Lee and Jeong Young Park**

Supplementary Information

Simultaneous Harvesting of Bipolar Plasmonic Hot Carriers for Boosting Photoconductivity in Ag Nanoprism-Coupled Lateral Si *p-n* Junction

Yujin Park,[1] Jihyang Park,[2,3] Yeonghoon Jin,[4] Yujin Roh,[1] Hyunhwa Lee,[1] Kyoungsik Yu,[4] Moonsang Lee,[2,3] and Jeong Young Park[1]*

[1]Department of Chemistry, Korea Advanced Institute of Science and Technology (KAIST), Yuseong-Gu, Daejeon, 34141, Republic of Korea

[2]Department of Materials Science and Engineering, Inha University, 100 Inha-ro, Michuhol-gu, Incheon, 22212, Republic of Korea

[3]Program in Semiconductor Convergence, Inha University, 100, Inha-ro, Michuhol-gu,

Incheon 22212, Republic of Korea

[4]School of Electrical Engineering, Korea Advanced Institute of Science and Technology

(KAIST), Yuseong-Gu, Daejeon, 34141, Republic of Korea

* Correspondence to mslee@inha.ac.kr, and jeongypark@kaist.ac.kr

Keywords: localized surface plasmon resonance, hot electrons, hot holes, Ag nanoprism, plasmonic photodetector

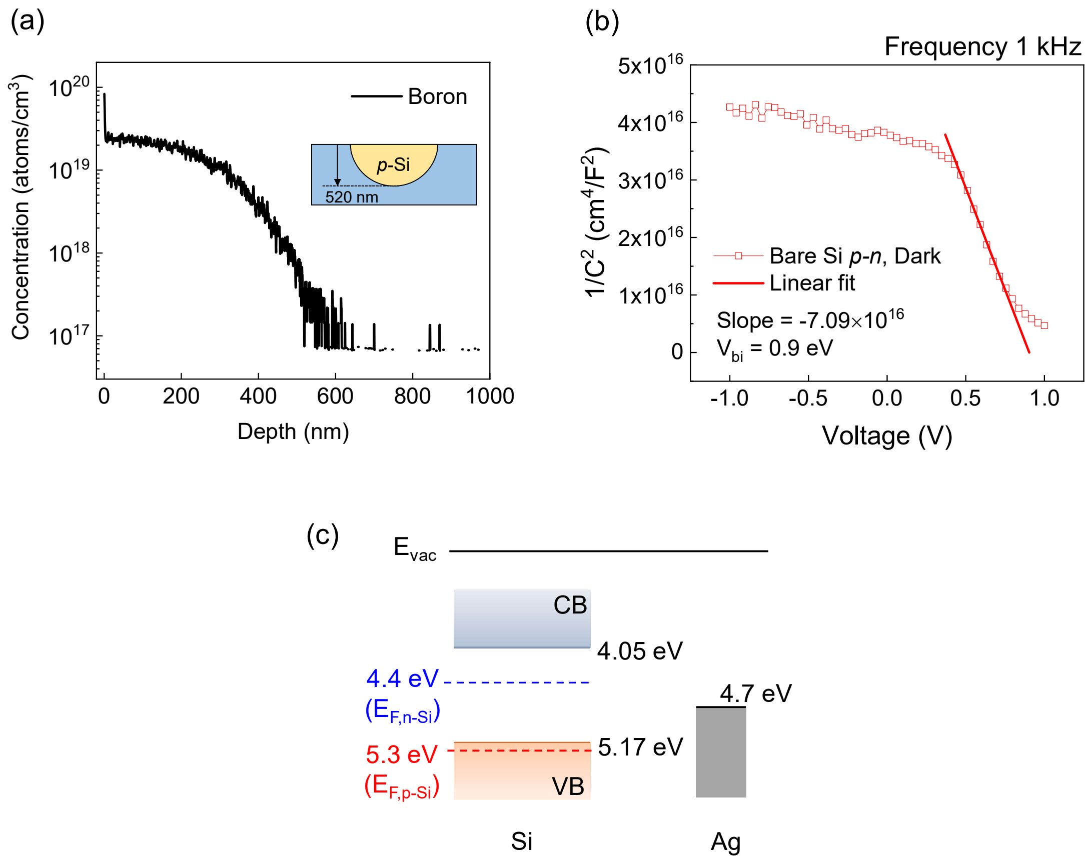

Figure S1. (a) Profile of boron concentration in *p*-Si. (b) Mott-Schottky plot of a bare Si *p*-*n* measured under dark conditions, with the AC bias frequency of 1 kHz. (c) Energy levels of *n*-type Si, *p*-type Si and Ag.

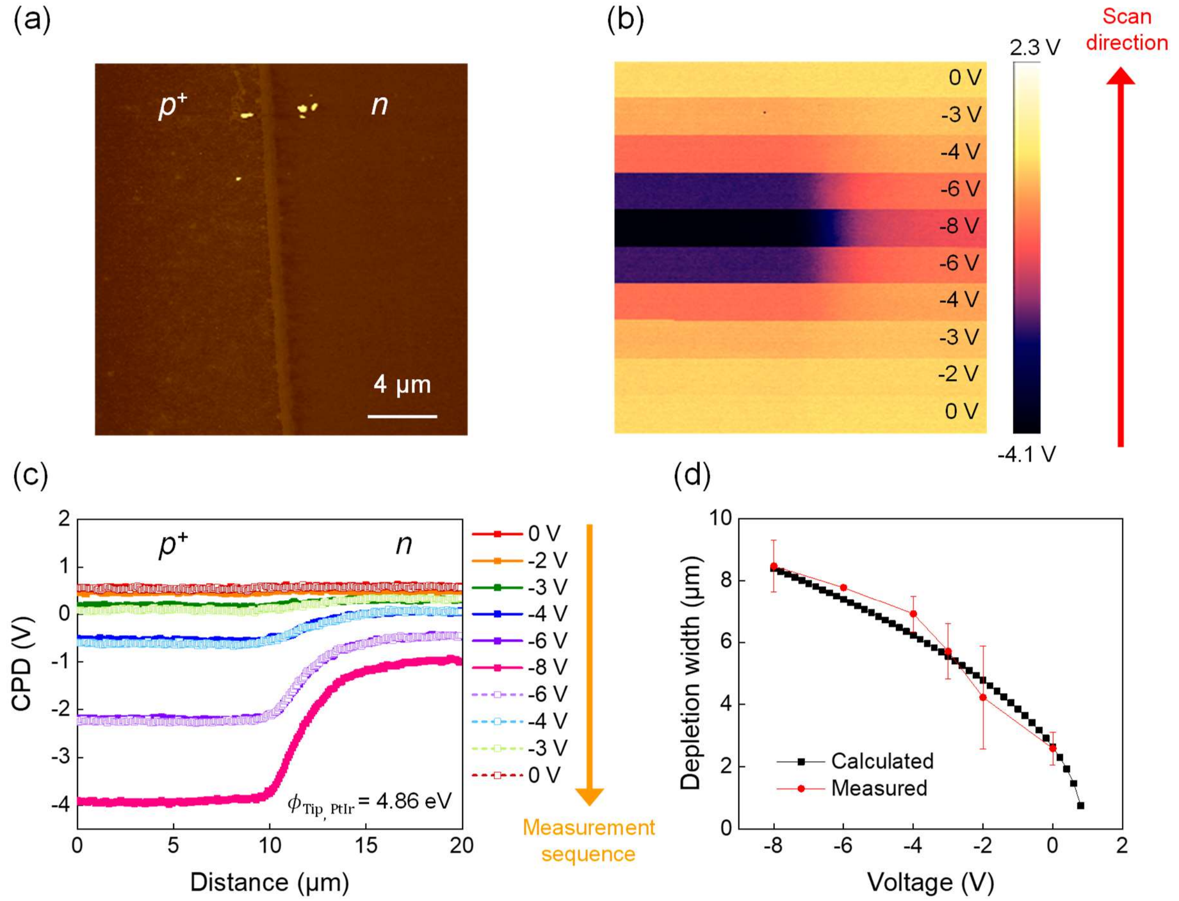

Figure S2. (a) Topography and (b) contact potential difference (CPD) mapping image of a bare Si *p-n* diode measured under varying external biases. The *p*-Si was biased from 0 V to –8 V, while the *n*-Si was grounded. The scan direction was from bottom to top. (c) CPD profiles obtained from the CPD mapping image of the bare Si *p-n*. (d) Comparison between theoretically calculated and experimentally measured depletion width results as a function of reverse bias on the bare Si *p-n* diode.

The results demonstrated that the surface potential gradient becomes increasingly pronounced toward the *n*-Si region as the reverse bias increased. By differentiating surface potential with respect to the distance, we acquired the *E*-field distribution of the bare lateral Si *p-n* junction. Since the internal *E*-field exists only at the depletion layer, we were able to experimentally evaluate the depletion width by measuring the distance of the area where the *E*-field is observed. Accordingly, the depletion width was found to be reversibly vary from 2 μm to 8 μm as the reverse bias was adjusted from 0 V to –8 V. Additionally, we theoretically calculated the depletion width based on doping concentration (See Supplementary Note 2), and confirmed the calculated values was in good agreement with the experimentally acquired values.

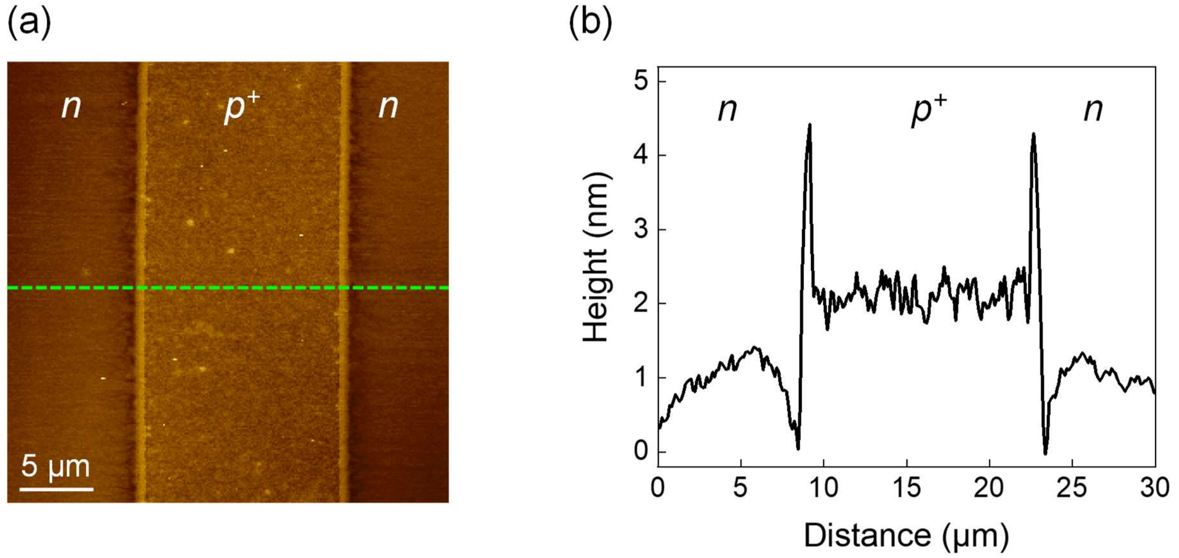

Figure S3. (a) Topography image and (b) height profile along the green line of a bare lateral Si *p-n* substrate.

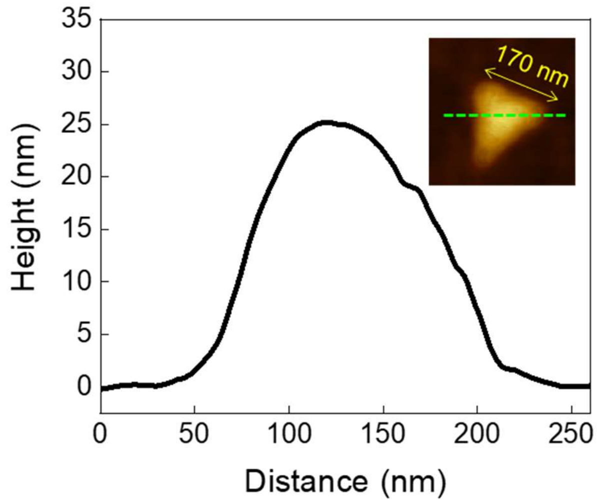

Figure S4. Height profile of a single Ag prism along the green line of the inset image. The inset image shows an AFM topographic image of an Ag prism on a Si substrate.

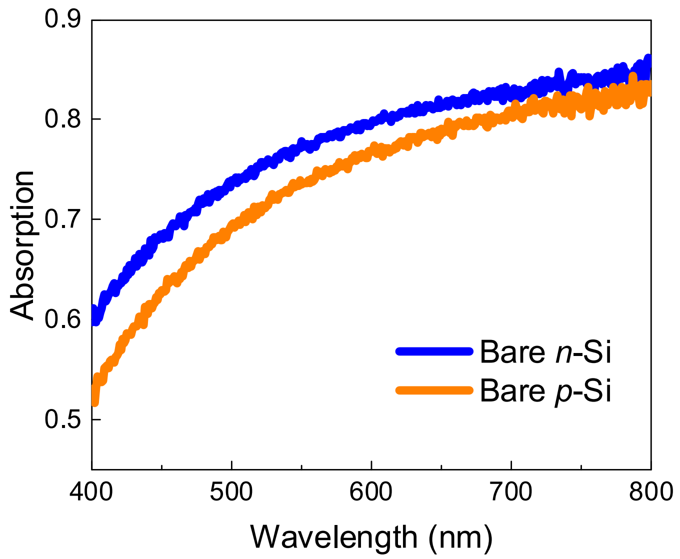

Figure S5. Absorption spectra of bare *n*-Si and bare *p*-Si substrates.

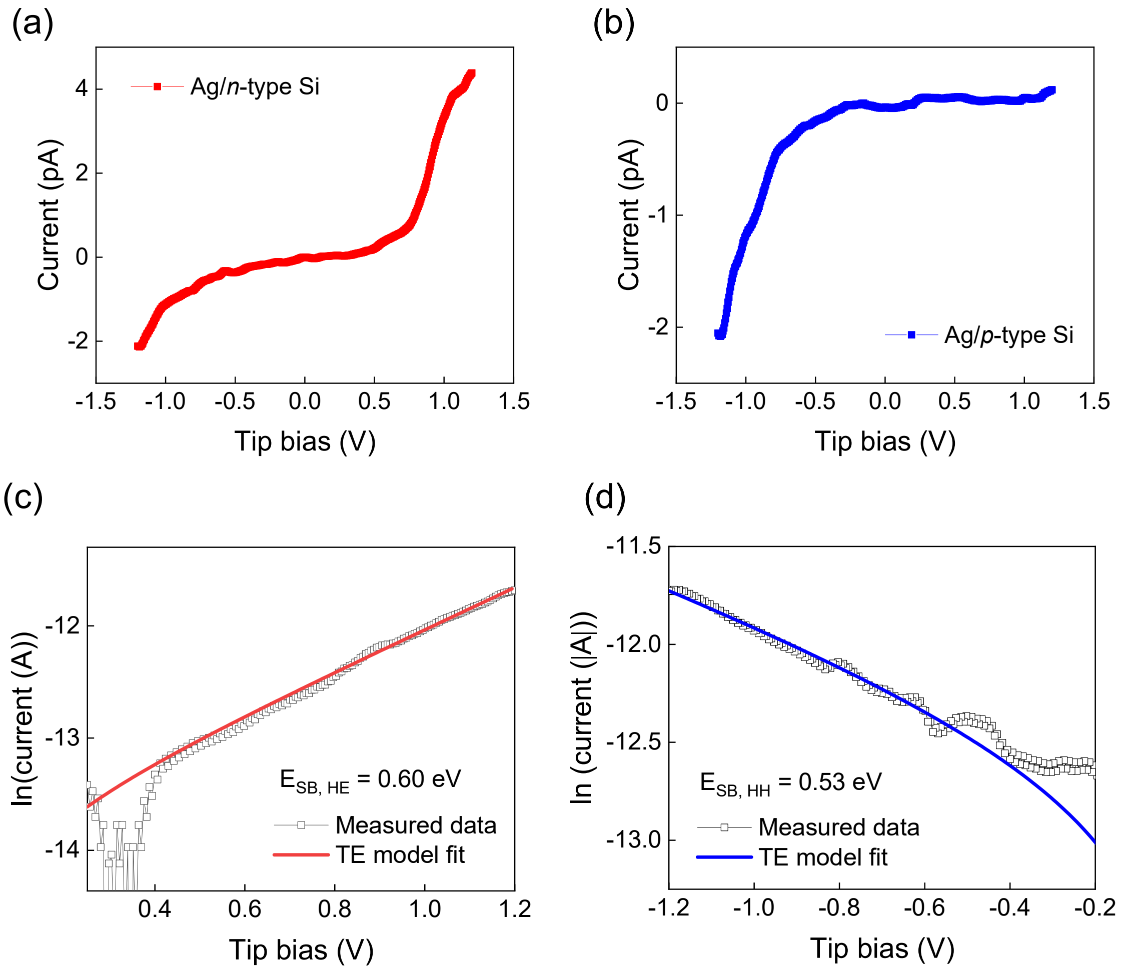

Figure S6. *I*–*V* curves measured on (a) the Ag/*n*-type Si structure and (b) the Ag/*p*-type Si using conductive AFM. The thermionic emission (TE) model fitted results of (c) the Ag/*n*-type Si structure and (d) the Ag/*p*-type Si. The E_{SB} on Ag/*n*-type Si and Ag/*p*-type Si are labeled as $E_{SB,\,HE}$ and $E_{SB,\,HH}$, respectively.

The Schottky barrier heights (E_{SB}) can be evaluated by fitting the acquired *I*–*V* curves to the thermionic emission model, which is given by:

$$I = AA^{**}T^{2}exp\left(-\frac{qE_{SB}}{k_bT}\right)\left[exp\left(\frac{q(V-IR_{ser})}{\eta k_bT}-1\right)\right] \qquad \text{Equation S1}$$

where A is the area of Schottky junction, A^{**} is the Richardson constant, q is the elementary charge, E_{SB} is the Schottky barrier height, k_b is the Boltzmann constant, T is the temperature, R_{ser} is the series resistance, and η is the ideality factor.

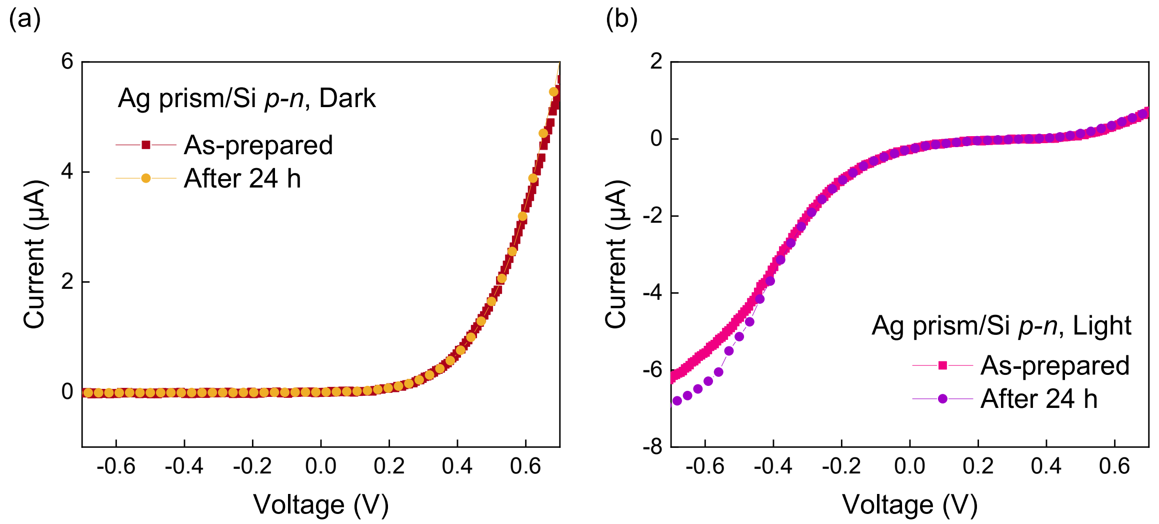

Figure S7. *I–V* curves measured immediately after fabrication and after 24 hours for Ag prism/Si *p-n* under (a) dark conditions and (b) light illumination. A tungsten-halogen lamp with an intensity of 9 mW/cm^2 was used as the light source.

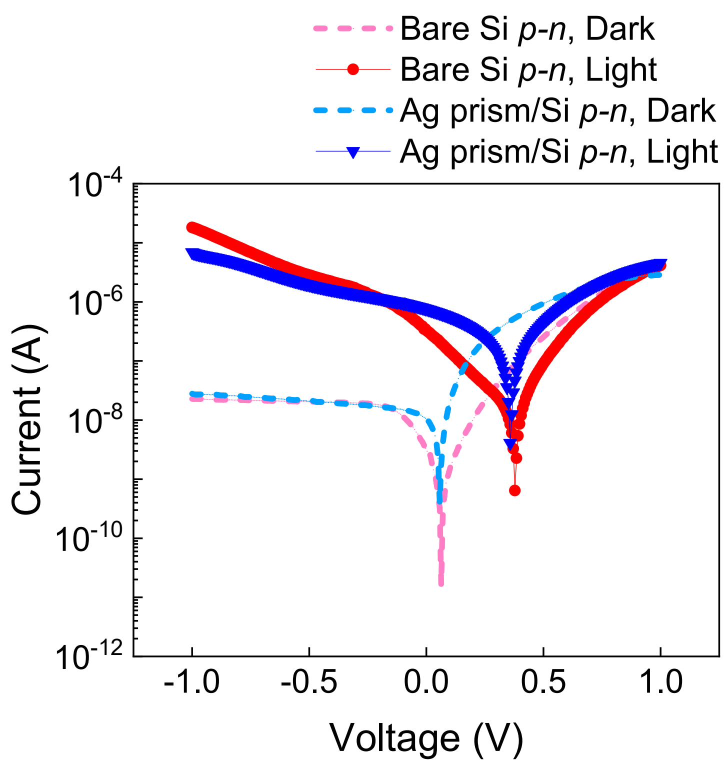

Figure S8. *I*–*V* curves measured on the bare Si *p-n* and the Ag prism/Si *p-n* with the Ag prism deposited area halved.

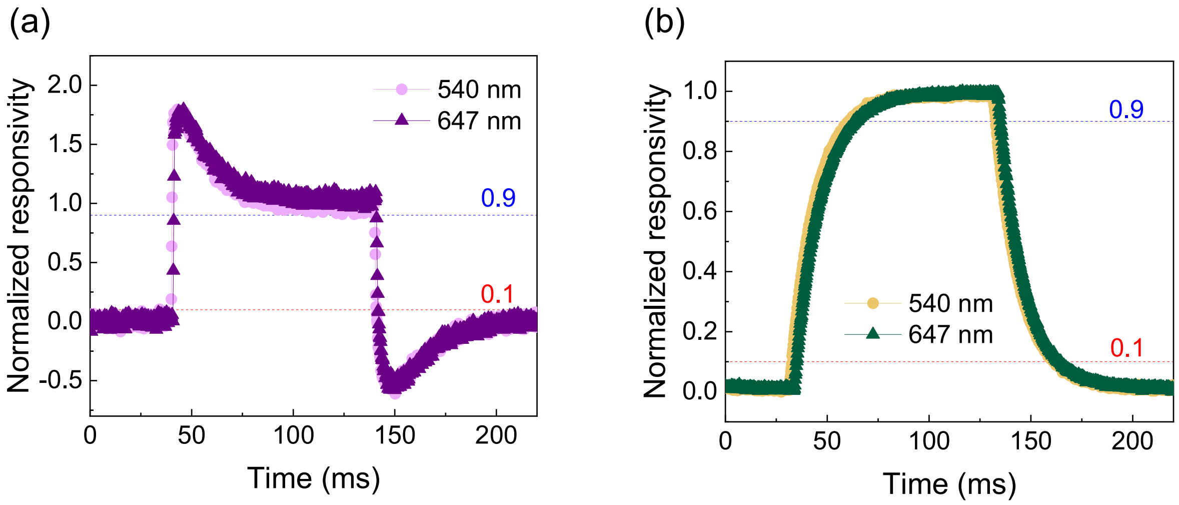

Figure S9. Normalized transient responsivity of (a) the bare Si *p-n* and (b) the Ag prism/Si *p-n* diode.

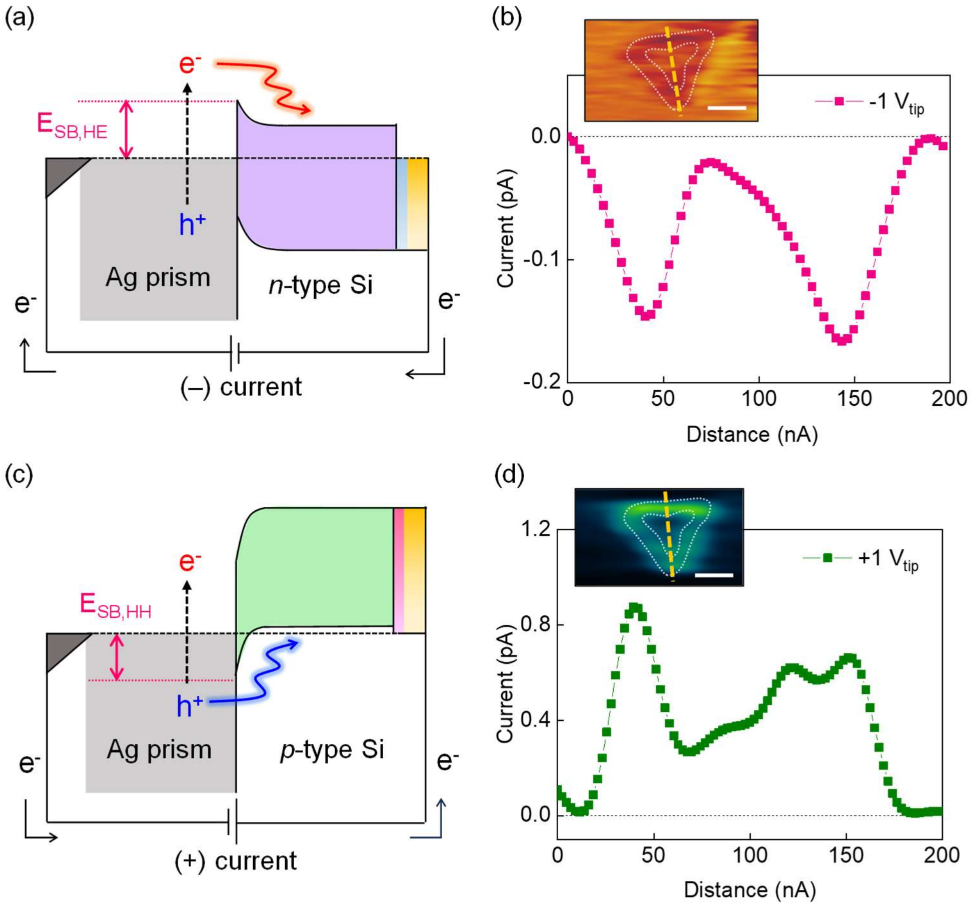

Figure S10. (a) Energy diagram illustrating photocurrent measurement on the Ag prism/*n*-Si using pc-AFM. (b) Photocurrent profile measured along the yellow dashed line shown in the inset. (c) Energy diagram illustrating photocurrent measurement on the Ag prism/*p*-Si using pc-AFM. (d) Photocurrent profile measured along the yellow dashed line shown in the inset. The scale bars are 80 nm.

In the Ag prism/*n*-Si structure (Figure S10a), hot electrons are injected into *n*-Si structure and flow toward the Ag prism through external circuit. This results in a negative photocurrent, which is more pronounced under reverse bias, due to the $E_{SB,HE}$ lowering that promotes hot-electron collection. Indeed, the photocurrent profile along the yellow dashed line in the photocurrent map of Ag prism on *n*-Si substrate demonstrates strong negative photocurrent peaks at the hot spots (edges and corners) of the Ag prism (Figure S10b). Conversely, in the Ag prism/*p*-Si structure, hot holes are collected in *p*-Si region (Figure S10c), resulting in a positive photocurrent at the hot spots (Figure S10d). These findings support our claim that hole holes are the primary contributor in *p*-Si region, while hot electrons are more significant in the *n*-Si region.

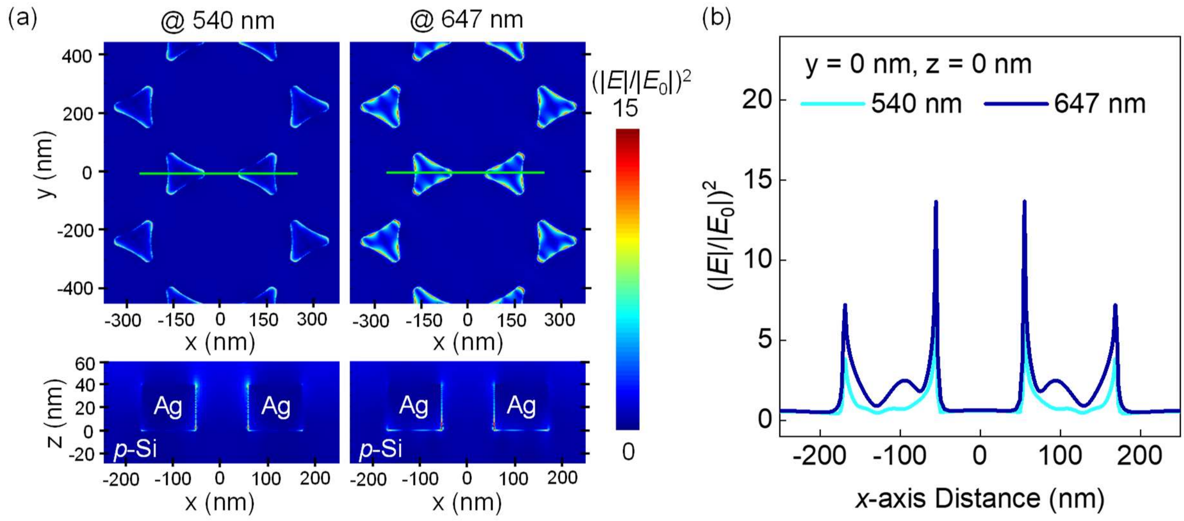

Figure S11. (a) FDTD simulations of E-field intensity $(|E|/|E_0|)^2$ on Ag prism/n-Si under 540 nm and 647 nm. Simulations are shown in a top view (upper panel) and a cross-sectional view along the green solid line in the top view image (lower panel). (b) E-field enhancement intensity profile along the x-axis at y=0 nm and z=0 nm of the Ag prism/p-Si structure.

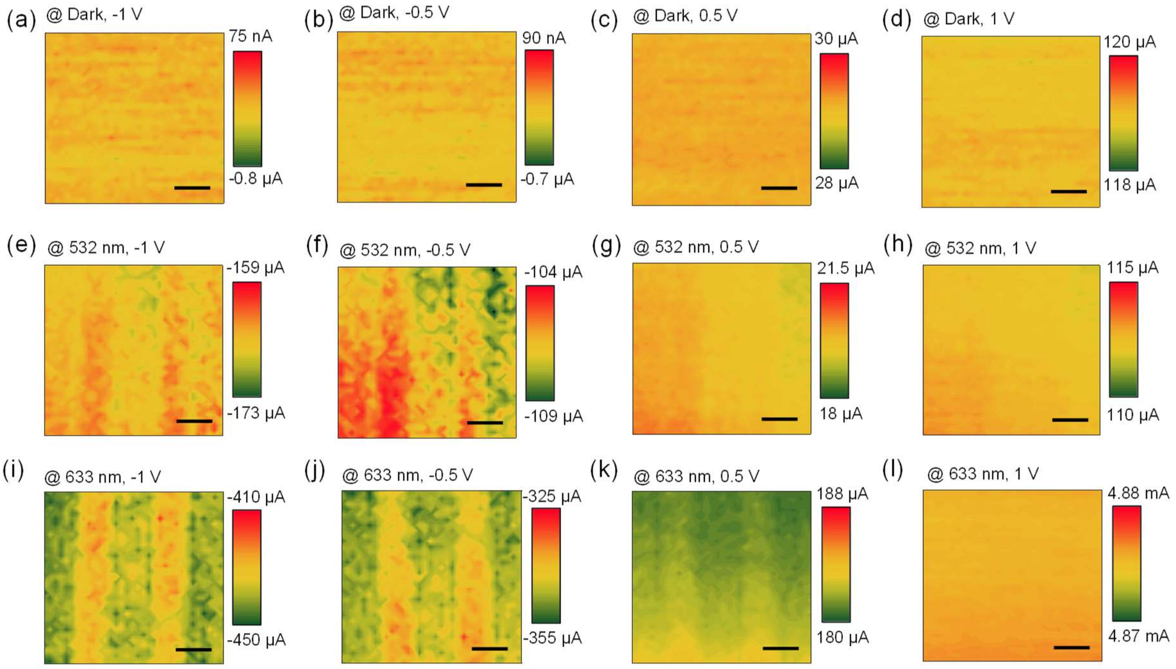

Figure S12. Scanning current mapping images of the Ag prism/Si *p-n* structure, excited with three different irradiation conditions: (a-d) dark, (e-h) 532 nm, and (i-l) 633 nm. The applied voltages for the images are (a,e,i) –1 V, (b,f,j) –0.5 V, (c,g,k) 0.5 V, and (d,h,l) 1 V. The scale bars in all images are 13 µm.

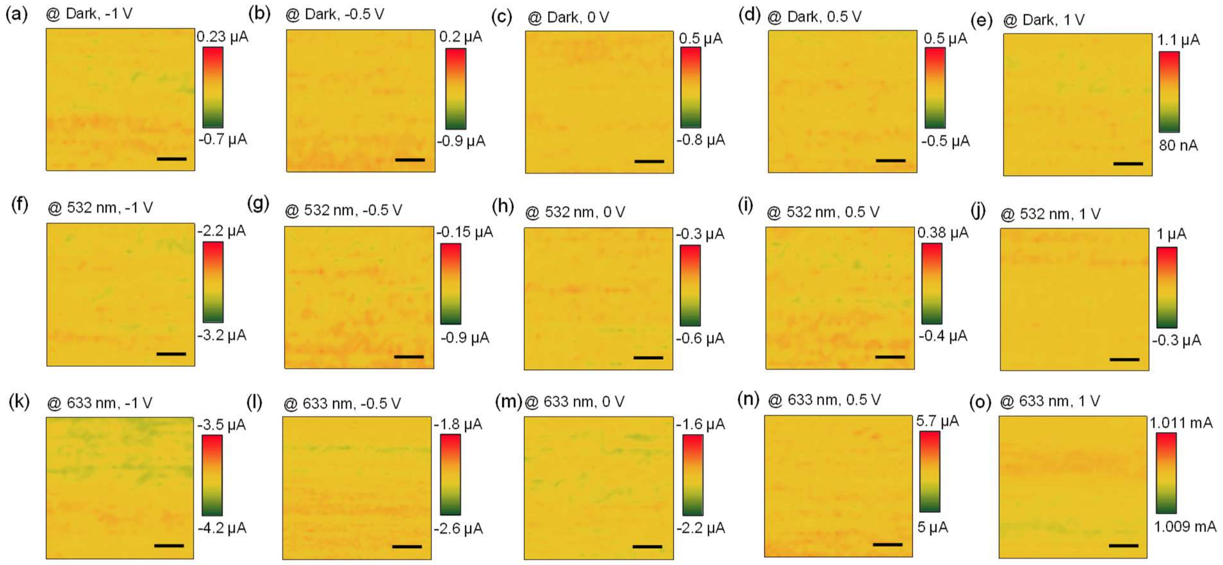

Figure S13. Scanning current mapping images of the bare Si *p-n* structures, excited with three different irradiation conditions: (a-e) dark, (f-j) 532 nm, and (k-o) 633 nm. The applied voltages for the images are (a,f,k) –1 V, (b,g,l) –0.5 V, (c,h,m) 0 V, (d,i,n) 0.5 V, and (e,j,o) 1 V. The scale bars in all images are 13 µm.

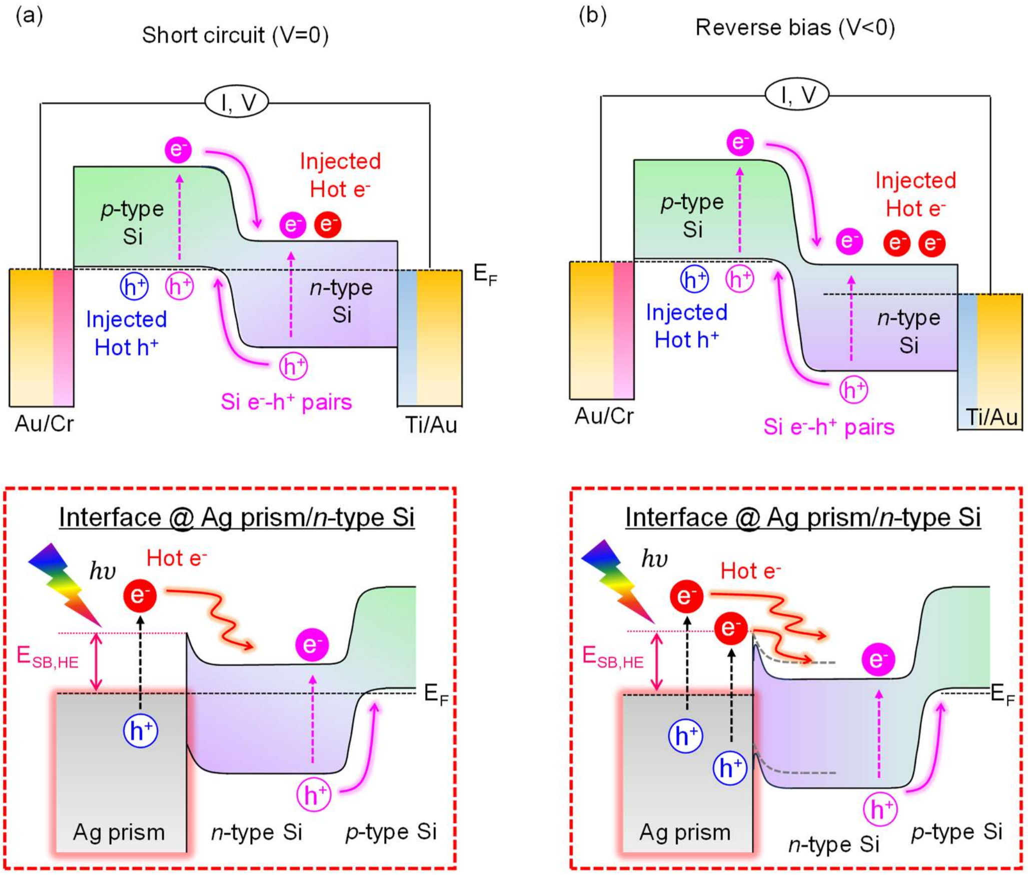

Figure S14. Schematic of the bipolar HC injection process in the Ag prism/Si *p-n* junction under (a) short circuit and (b) reverse bias. The upper images show the mechanism of photocurrent generation in the lateral Si *p-n* junction under short circuit and reverse bias, while the lower images depict the corresponding hot-electron injection process at Ag prism/*n*-Si. The banding at Ag prism/*n*-Si interface under short-circuit conditions is overlaid as the grey dashed line in Figure S14b, for comparison.

Macroscopic scanning current maps show increased photocurrent contrast between the *n*-Si and *p*-Si regions with higher reverse bias on the Ag prism/Si *p-n* (Figure 5), while the contrast was not observed in a bare Si *p-n* structure (Figure S13). This effect is attributed to the enhanced differences in HC collection between hot holes in *p*-Si and hot electrons in *n*-Si. Particularly, *p*-Si was grounded during the measurement, causing the reverse bias to shift the energy levels of *n*-Si to the lower levels, while maintaining the energy levels of *p*-Si. Since the Fermi level of Ag nanoprisms is fixed due to the Fermi-level pinning,[1] the applied reverse bias effectively lowers the $E_{SB,HE}$, induced by image forces in n-Si substrate, as shown in the lower image of Figure S14b.[2-3] This reduction of E_{SB} under reverse bias in Ag prism/Si *p-n* promotes hot-electron collection in *n*-Si and maintains consistent hot-hole collection in *p*-Si, resulting in increased photocurrent contrast.

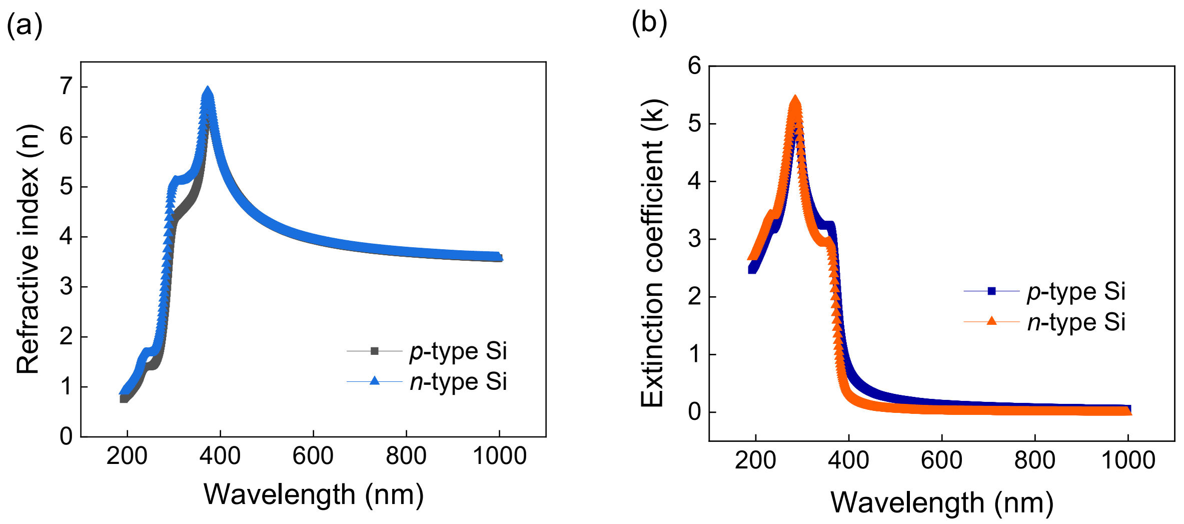

Figure S15. (a) Refractive index and (b) extinction coefficient as a function of wavelength of a *p*-type Si, an *n*-type Si.

Table S1. Summarization of rise and fall time measured on the bare Si *p-n* and the Ag prism/Si *p-n* photodiodes

	540 nm		647 nm	
	Rise time (ms)	Fall time (ms)	Rise time (ms)	Fall time (ms)
Bare Si *p-n*	0.4	0.8	0.4	0.8
Ag prism/Si *p-n*	28.6	28.8	29	28.9

Supplementary Note 1. Estimation of Built-in Potential and Doping Concentration, and Work Function of Si Using the Mott-Schottky Equation

The Mott-Schottky equation can be defined as the following equation:

$$\frac{1}{C^2} = \frac{2}{q\varepsilon\varepsilon_0}\left(\frac{1}{N_{A,avg}} + \frac{1}{N_{D,avg}}\right)(V_{bi} - V) \qquad \text{Equation S2}$$

where C is the capacitance, q is the elementary charge, ε is the relative permittivity of Si, ε_0 is the vacuum permittivity, V_{bi} is the built-in potential, and $N_{A,avg}$ and $N_{D,avg}$ are the averaged doping concentration of p-Si and n-Si, respectively. The doping concentration of the p-Si was determined by analyzing time-of-flight secondary ion mass spectrometry (TOF-SIMS) (Figure S1a), and the $N_{A,avg}$ was estimated 1.3×10^{19} cm^{-3}. V_{bi} can be obtained by fitting the linear region in the Mott-Schottky plot, where the value of the intercept with x-axis corresponds to V_{bi}. Similarly, $N_{D,avg}$ can be derived using the following equation:

$$N_{D,avg} = \left[-\frac{q\varepsilon\varepsilon_0}{2}\left(\frac{d\left(\frac{1}{C^2}\right)}{dV}\right) - \frac{1}{N_{A,avg}}\right]^{-1} \qquad \text{Equation S3}$$

The calculated V_{bi} and $N_{D,avg}$ are 0.9 eV and 1.7×10^{14} cm^{-3}, respectively. Accordingly, the Fermi level of n-Si and that of p-Si can be derived by the following equation.

$$E_{F,n} - E_i = kT\ln\left(\frac{N_{D,avg}}{n_i}\right) \qquad \text{Equation S4}$$

$$E_{F,p} = E_{F,n} + V_{bi} \qquad \text{Equation S5}$$

where $E_{F,n}$ is the Fermi level of n-Si, $E_{F,p}$ is the Fermi level of p-Si, E_i is the intrinsic Fermi level of Si, k is the Boltzmann constant, T is the temperature, and n_i is the intrinsic carrier concentration of Si. Accordingly, $E_{F,n}$ and $E_{F,p}$ are estimated to be 4.4 eV and 5.3 eV, respectively.

Supplementary Note 2. Calculation of Depletion Width

The depletion width (w) of the lateral Si *p-n* junction can be defined as the following equation:

$$w = w_n + w_p \quad \text{Equation S6}$$

where w_n and w_p are the depletion width at *n*-Si and *p*-Si, respectively. However, since *p*-Si is degenerated, we can simplify Equation S6 as the following equation:

$$w = w_n + w_p \approx w_n \quad \text{Equation S7}$$

$$w \approx w_n = \sqrt{\frac{2\varepsilon\varepsilon_0(V_{bi} - V_a)}{qN_{D,avg}}} \quad \text{Equation S8}$$

where V_a is the applied bias.

Supplementary Note 3. Calculation of Responsivity and Detectivity

The photoresponse characteristics, including responsivity and detectivity, can be calculated using the following equations:

$$\mathrm{I_{photo}} = \mathrm{I_L} - \mathrm{I_D} \qquad \text{Equation S9}$$

$$\mathrm{R} = \frac{\mathrm{I_{photo}}}{\mathrm{A \times P}} \qquad \text{Equation S10}$$

$$D^* = \frac{R \times A^{0.5}}{(2 \times q \times I_D)^{0.5}} \qquad \text{Equation S11}$$

where I_D and I_L indicate the currents in dark and under light, respectively, I_{photo} denotes the difference between current under light and dark conditions, P explains light power, A is active area of the diodes, and q is elementary charge.

Supplementary Note 4. Estimation of quantum efficiency (QE) of hot carriers in Ag prisms and that of direct excitation (DE) in Si *p-n* junction

The photocurrent (I_{ph}) measured on the Ag prism/lateral Si *p-n* junction can be defined as the following equation:

$$I_{ph} = I_{DE,Si\,p-n} + I_{HC,Ag\,prisms} \quad \text{Equation S12}$$

where $I_{DE,Si\,p-n}$ and $I_{HC,Ag\,prisms}$ are the photocurrent governed by the direct excitation (DE) in Si *p-n* junction and the hot carriers (HC) in Ag prisms, respectively. Assuming that one absorbed photon in Si generates a single e–h pair, $I_{DE,Si\,p-n}$ can be defined as the following equation:

$$I_{DE,Si\,p-n} = I_{DE,n-Si} + I_{DE,p-Si} \quad \text{Equation S13}$$

where $I_{DE,n-Si}$ and $I_{DE,p-Si}$ are the photocurrent created by the DE in the *n*-Si region and *p*-Si region, respectively, within the lateral Si *p-n* junction. Considering $N_{A,avg}$ = 1.7×10^{14} cm^{-3} and $N_{A,avg}$= 1.3×10^{19} cm^{-3}, the diffusion length of minority carriers in *n*-Si and *p*-Si is approximated to 600 μm and 2 μm, respectively.[4-5] We evaluated depletion width of the Si *p-n* junction in this study to be 2.6 μm. Thus, Equation S13 can be simplified to the following equation:

$$I_{DE,Si\,p-n} \approx I_{DE,n-Si} \quad \text{Equation S14}$$

$$\begin{aligned} I_{DE,n-Si} &= q \times C \times (Number\ of\ absorbed\ photons) \\ &= q \times C \times P_{inc}(\lambda) \times Abs_{n-Si}(\lambda) \times \frac{1\ \text{eV}}{1.6 \times 10^{-19}\ \text{J}} \\ &\times \frac{\lambda}{1240\ \text{eV}} \end{aligned} \quad \text{Equation S15}$$

where C is the proportionality coefficient, $P_{inc}(\lambda)$ is the power of incident light, $Abs_{n-Si}(\lambda)$ is the absorption of *n*-Si, and λ is the wavelength. Therefore, the QE of DE in the Si *p-n* junction can be expressed as the following equation:

$$QE_{DE,Si\,p-n} \approx QE_{DE,n-Si} \quad \text{Equation S16}$$

$$QE_{DE,n-Si} = R_{DE,\,n-Si} \times \frac{1240\ \text{eV}}{\lambda} = \frac{I_{DE,n-Si}}{P_{inc}(\lambda)} \times \frac{1240\ \text{eV}}{\lambda} = C \times Abs_{n-Si}(\lambda) \quad \text{Equation S17}$$

where $R_{DE,\,n-Si}$ is the responsivity of DE in n-Si. $Abs_{n-Si}(\lambda)$ is estimated using the FDTD method. We refer to preliminary literature to determine the proportionality constant C.[6] We assume C to be independent of the wavelength and its value is determined from the requirement that $QE_{DE,n-Si}$ at 800 nm matches the experimentally measured QE on the Ag prism/Si *p-n* junction, where the absorption of Ag prisms is negligible at 800 nm. Finally, $QE_{HC,\,Ag\,prisms}$ is acquired by subtracting $QE_{DE,n-Si}$ from the experimentally measured QE on the Ag prism/Si *p-n* junction.

References

[1] R. Islam, G. Shine, K. C. Saraswat. *Appl. Phys. Lett.* 2014, ***105***, 182103.
[2] H. Lee, H. Lee, J. Y. Park. *Nano Lett.* 2019, ***19***, 891.
[3] H. Lee, K. Song, M. Lee, J. Y. Park. *Adv. Sci.* 2020, ***7***, 2001148.
[4] P. P. Altermatt, A. Schenk, F. Geelhaar, G. Heiser. *J. Appl. Phys.* 2003, ***93***, 1598.
[5] P. P. Altermatt, J. O. Schumacher, A. Cuevas, M. J. Kerr, S. W. Glunz, R. R. King, G. Heiser, A. Schenk. *J. Appl. Phys.* 2002, ***92***, 3187.
[6] Z. Fang, Z. Liu, Y. Wang, P. Ajayan, P. Nordlander, N. J. Halas. *Nano Lett.* 2012, ***12***, 3808.
